# MARAS: Mutual Authentication and Role-Based Authorization Scheme for Lightweight Internet of Things Applications

**DOI:** 10.3390/s23125674

**Published:** 2023-06-17

**Authors:** Özlem Şeker, Gökhan Dalkılıç, Umut Can Çabuk

**Affiliations:** 1 Graduate School of Natural and Applied Sciences, Dokuz Eylul University, Izmir 35390, Turkey; ozlem.yerlikaya@cs.deu.edu.tr; 2 Department of Computer Engineering, Dokuz Eylul University, Izmir 35390, Turkey; dalkilic@cs.deu.edu.tr; 3 Department of Electrical & Computer Engineering, San Diego State University, San Diego, CA 92182, USA

**Keywords:** authorization, HOTP, IoT, MQTT, mutual authentication

## Abstract

The Internet of things (IoT) accommodates lightweight sensor/actuator devices with limited resources; hence, more efficient methods for known challenges are sought after. Message queue telemetry transport (MQTT) is a publish/subscribe-based protocol that allows resource-efficient communication among clients, so-called brokers, and servers. However, it lacks viable security features beyond username/password checks, yet transport-layer security (TLS/HTTPS) is not efficient for constrained devices. MQTT also lacks mutual authentication among clients and brokers. To address the issue, we developed a mutual authentication and role-based authorization scheme for lightweight Internet of things applications (MARAS). It brings mutual authentication and authorization to the network via dynamic access tokens, hash-based message authentication code (HMAC)-based one-time passwords (HOTP), advanced encryption standard (AES), hash chains, and a trusted server running OAuth2.0 along with MQTT. MARAS merely modifies “publish” and “connect” messages among 14 message types of MQTT. Its overhead to “publish” messages is 49 bytes, and to “connect” messages is 127 bytes. Our proof-of-concept showed that the overall data traffic with MARAS remains lower than double the traffic without it, because “publish” messages are the most common. Nevertheless, tests showed that round-trip times for a “connect” message (and its “ack”) are delayed less than a percentile of a millisecond; for a “publish” message, the delays depend on the size and frequency of published information, but we can safely say that the delay is upper bounded by 163% of the network defaults. So, the scheme’s overhead to the network is tolerable. Our comparison with similar works shows that while our communication overhead is similar, MARAS offers better computational performance as it offloads computationally intensive operations to the broker side.

## 1. Introduction

Many electronic devices containing sensors/actuators are integrated with networking capabilities to enable communications and enhanced automation. Many such devices can also establish Internet connections, resulting in the emergence of the Internet of things (IoT) concept. The devices that constitute the IoT may include sensors, actuators, interfaces, robotics, and storage. They gather, generate, store, and disseminate data and further exchange them with other devices connected to the internet. They may even manage themselves with no or little human interaction. Wireless sensor networks (WSNs) [1], the premise of the IoT, may include a large number of sensor nodes that are low-cost, small-sized, and have limited bandwidth, so they can communicate over specific lightweight protocols. Many modern IoT deployments include devices, functionalities, and goals similar to that of a WSN. This is why they also have similar constraints, although they support the Internet Protocol (IP) to some extent [2,3,4].

The number of devices connected to the Internet is dramatically increasing, thanks to the IoT and its industrial applications. Examples of current research topics include the relation between energy availability, sensing quality, and the overall throughput in cognitive radio networks powered by energy harvesting techniques [5] and maximizing packet collection performance in wireless-powered IoT networks [6]. Those highlight the significance of efficient energy management and data transmission strategies in IoT networks. On the other hand, IoT devices with limited bandwidth, memory, and computing capabilities are also vulnerable to security threats. Due to their limitations, traditional security mechanisms used in computers are unlikely to be implemented in IoT devices. In addition, the sensor nodes can reach out to the Internet via some gateways running on the application layer [7]. Therefore, the gateways need to be designed to interpret many different protocols. For the gateways and other intermediary elements, this poses many design challenges. Message queue telemetry transport (MQTT) (no longer accepted as an acronym since version 5.0) is a lightweight communication protocol widely adopted in IoT applications due to its resource efficiency, flexibility, and scalability [8]. However, it lacks advanced security measures that are highly desired in critical applications. Within the scope of this study, we propose a security mechanism to provide mutual authentication and role-based authorization for the nodes in an IoT ecosystem running the MQTT protocol.

The constrained application protocol (CoAP), an alternative to MQTT, is also popular in IoT. It utilizes the user datagram protocol (UDP) [9] and is comparable to the hypertext transfer protocol (HTTP) since it is a web-based scheme. It provides reduced packet header sizes for resource-constraint devices. However, the CoAP header size is four bytes, whereas the MQTT header size is only two bytes [10]. Moreover, MQTT reportedly demonstrates better performance than CoAP in terms of packet losses and transmission latencies under increased network traffic [11]. Hence, MQTT is apparently more “lightweight”, and thus, it is preferred over CoAP in this study. Before proceeding to the details of the problem statement and our novel contributions, we provide some background regarding MQTT and other referred concepts.

### 1.1. Background

An introduction to the MQTT protocol and its security features is provided in this part. There is also a brief discussion on using a hash-based message authentication code (HMAC)-based one-time password (HOTP).

#### 1.1.1. Overview of MQTT

The MQTT protocol was developed by IBM in 1999 and was standardized by the organization for the advancement of structured information standards (OASIS) in 2013 [12] to provide lightweight machine-type communications. Thanks to its publish/subscribe-based architecture, MQTT is a convenient communication protocol for most IoT scenarios. It is preferred for devices with limited resources [13,14,15].

Nodes in an MQTT-driven network may have different roles. The clients (i.e., mostly sensor/actuator devices) can either be publishers or subscribers for any information flow (e.g., sensed temperature data). Such flows of information are tagged with topic labels (called a “topic”) considering their context (e.g., temperature). Hence, some clients may subscribe to certain topics and discard the rest.

There are also intermediary nodes, called brokers, responsible for distributing the information disseminated by the publishers to the interested subscribers. The broker keeps an up-to-date record of which client is interested in which topics. So, it shall forward the incoming data to the subscribers of that topic by filtering out the access rights. The publishers and subscribers do not have prior knowledge of each other. All the communication is carried out through the broker(s). A publisher and subscriber(s) do not have to be connected to the same network simultaneously [16]. Occasionally, a broker may also be a client at the same time [17]. Likewise, a publisher of one (or more) arbitrary topics may be a subscriber of others. Other types of nodes (e.g., servers, databases, etc.) may optionally co-exist in the ecosystem.

MQTT’s message packets include three components: a mandatory 2-byte fixed-length header, an optional variable-length header, and an optional payload. The fixed-size header consists of the control field that contains the 4-bit message type field, the 4-bit control flag, and the packet length field. MQTT supports 14 unique message types that are connection request (*CONNECT*), connection acknowledgment (*CONNACK*), subscription request (*SUBSCRIBE*), subscription acknowledgment (*SUBACK*), unsubscription request (*UNSUBSCRIBE*), unsubscription acknowledgment (*UNSUBACK*), publish data (*PUBLISH*), publish acknowledgment (*PUBACK*), publish received (*PUBREC*), publish released (*PUBREL*), publish complete (*PUBCOMP*), ping request (*PINGREQ*), ping response (*PINGRESP*) and disconnection request (*DISCONNECT*). Section 4 further explains these message types and their control flags.

#### 1.1.2. Security Features in MQTT

Due to its lightness, there is no advanced security mechanism built into the MQTT protocol, except for a basic authentication scheme involving a username and password- control over a user database. When there are no resource-constrained devices in the network, the transport layer security (TLS) protocol can also be used with MQTT. It is the most secure way to provide data confidentiality and integrity [18]. However, this may not be applicable if lightweight devices are included, because allocating a secure end-to-end channel via TLS requires complex and heavy computations. If no security mechanism is implemented along with MQTT, then the subscribers may grant access to all the messages.

Moreover, a malicious publisher may act as an attacker and may cause a denial of service (DoS) attack. The use of access token(s) was already recommended to provide authentication and authorization mechanisms in the existing literature [19], but there is no viable security mechanism between the clients and an authorization server, except for the TLS-based hypertext transfer protocol secure (HTTPS). HTTPS allows using tokens to manage access control [16]. Furthermore, no prior studies have yet suggested a client-side authentication of the broker(s), which is necessary to achieve mutual trust and security within the ecosystem. This study provides a two-step mutual authentication and authorization mechanism using a “trusted” third-party authentication server that runs OAuth 2.0 [20]. It is also challenging to manage role-based access controls due to the potentially high numbers of connected devices. Since MQTT has no native security mechanism to address these issues, a dynamic token scheme that includes the scopes (i.e., authorized topics) of the clients is considered via the OAuth 2.0 protocol [21].

#### 1.1.3. Using HOTP with MQTT

An authorization server (as discussed above), HOTP [22], and hash chains can be used to verify the identities of the client nodes and the broker(s) communicating via the MQTT protocol. HOTP already became a standard in 2005 [23] and is used to provide mutual authentication, as well as to prevent replay attacks without requiring time synchronization [24,25]. In the background, it relies on the HMAC, a unique secret (K), and also a counter value (C) that must be kept synchronized with the HOTP generator (and validator). A HOTP is generated through the truncation of a pre-computed HMAC-SHA-1 value, as shown below:

HOTP (K, C) = Truncate (HMAC_SHA-1 (K, C))(1)

The broker implements the HOTP generator role by combining the HOTP with Lamport’s backward hash chain scheme [22]. Nonetheless, since the exponentiation of such hash functions needs exhaustive computations on the clients (that are presumed to be constrained devices), a critical problem arises with Lamport’s original scheme. A feasible solution to that issue is to move the computationally heavy phases of the scheme to the brokers. Therefore, the hash chain is generated N-times by the broker itself, whereas the clients act as the HOTP validators and merely store the hash chain values [26]. The clients validate the broker while N decrements until reaching zero. They have (HOTP (client ID, 3)) value; ‘3’ is considered due to the handshaking procedure. The broker generates the HOTP hash chain value N-times in total, as given below, where h is the hashing function

p = h (h (… h (HOTP (clientID, 3)) …))(2)

Last but not least, the advanced encryption standard (AES) [27] (or equivalent) is required to provide data confidentiality in the links between the clients and the broker(s). In this way, subsistent data integrity can be offered, too.

### 1.2. Problem Statement

Concisely listed below are the main problems that this work addresses. They have emerged from the special requirements of lightweight IoT applications and the insufficiencies of the popular MQTT protocol.

- The MQTT protocol does not natively allow the client nodes to authenticate the brokers and vice versa;
- The MQTT protocol does not natively provide topic-based and role-based authorization. So, it is not possible to allow nor disallow certain clients to publish on or subscribe to particular topics;
- The MQTT protocol does not natively enforce data encryption and integrity measures;
- Lightweight IoT applications require resource-efficient security features while not degrading the overall performance.

The symbols and abbreviations used to describe the problem and the solution are introduced in Table 1.

### 1.3. Contributions

The distinguished contributions of our work are discussed below:

- A novel method that adds mutual authentication and role-based authorization features to the MQTT protocol;
- To the best of our knowledge, the proposed method is the only one that grants mutual authentication among the MQTT clients and the broker(s) without relying on a secure channel (e.g., HTTPS);
- In addition to the two-factor mutual authentication, there is a role- and topic-based authorization over a trusted third-party server that enforces AES-encrypted messaging. These features altogether result in an exceptionally complete security solution for MQTT;
- The method, when applied, does not affect the performance of the system significantly. It only causes networking delays that are a tiny fraction of a millisecond. That largely distinguishes our work from those implementing TLS/HTTPS between clients and brokers (or servers).

Our solution can be seen as a protocol extension or a method that can be applied on top of MQTT. In the simplest terms, the scheme can be summarized as follows: First, a developer sets up the authorization server by recording the nodes and the roles/topics in which they are allowed to participate. The broker authenticates a client who requests to publish on or subscribe to a topic using a pre-shared secret and HOTP. Likewise, the client also authenticates the broker. Later, the broker checks the authorization server to assure that the requesting client has the right to do what it wants. If authentication and authorization are completed successfully, encrypted communication starts between the parties.

We analyzed the security vulnerabilities of Fremantle et al.’s scheme [21] and improved it to achieve satisfactory security features including authorization and mutual authentication for all nodes and brokers of MQTT. In the study, they suggested using OAuth 2.0 on an 8-bit Arduino Uno device. The connection request is made by including an access token as a plain text username in the MQTT connect message. Due to the limited memory and processing capacity of Arduino Uno, they claimed that generating a refresh token was not feasible. However, this implementation introduces potential security vulnerabilities. An attacker may intercept and listen to the access tokens transmitted as clear text, compromising the security of the system. Additionally, the attacker can generate message traffic and consume the device’s energy by continuously flooding connection requests using a constant token. To address these security concerns, MARAS incorporates a second authentication step. In situations where static plain text must be used, MARAS employs HMAC-based one-time passwords. Following successful connections, the HMAC-based OTP is refreshed, ensuring that the authentication mechanism remains dynamic and secure. By introducing this extra layer of security, MARAS mitigates the risks associated with using static plain text tokens and strengthens the overall security of the system.

Furthermore, in the described scenario, where the access token is transmitted as plain text and the attacker has the ability to intercept and reveal the token, there is a risk of device spoofing. An attacker could potentially impersonate the device and transmit unauthorized or inappropriate messages to the MQTT broker. Such unauthorized message transmissions can lead to various security and integrity concerns within the system. It can compromise the confidentiality and privacy of the transmitted data, manipulate the control and operation of the energy management system, or even cause disruptions and malicious actions within the network. To mitigate the risk of device spoofing and the transmission of inappropriate messages, MARAS implemented additional security measures including the following:

- Device authentication: Implemented with a two-step mutual authentication mechanism combining a token-based approach and HOTP with a hash chain, where both a client and an MQTT broker authenticate each other to ensure the legitimacy of the communication;
- Confidentiality: By employing hardware-powered AES encryption capabilities provided by devices like ESP32, the payload encryption enhances the confidentiality of the messages transmitted through MQTT. This added layer of security protects sensitive data from unauthorized access or interception;
- The implementation of the OAuth 2.0 protocol in the existing study has played a crucial role in ensuring that all authenticated devices within the application can send and receive messages only pertaining to the topic they are associated with. This authorization mechanism ensures that devices have the necessary permissions to publish or subscribe to specific topics;
- Considering the input and output capabilities of IoT devices, it is essential to have a secure and efficient authorization mechanism in place. The OAuth 2.0 protocol aligns well with the requirements of IoT devices, as it offers a scalable and flexible approach to managing access rights and permissions.

## 2. Related Works

This section discusses the previous efforts on securing the MQTT protocol by integrating authentication and authorization solutions. The effectiveness of the related works is elaborated on, and unresolved issues are pointed out. Fremantle et al. [21] integrated the OAuth 2.0 protocol to provide means of access control to the MQTT communications. They claim that conventional authorization and authentication systems are not suitable for IoT scenarios because it is difficult to manage each device using a username–password pair and related role credentials, but they did not provide any user interface or interaction scheme between the machine(s) and the developer. The access token and the scope(s) are obtained by each client using the OAuth 2.0 protocol, that is, to manage the authentication and authorization mechanisms whenever a client connects to the MQTT broker. The MQTT protocol runs over the transmission control protocol (TCP), so it can utilize TLS to provide a secure channel in between, but most IoT devices are limited in terms of battery, storage, and computational power. Likewise, in their work, the connection between the clients and the MQTT broker is established without TLS due to using an 8-bit Arduino as the client device. Refreshing the token is challenging because of its limited storage. However, merely using the same token leads to security vulnerabilities such as eavesdropping, spoofing, and denial of service attacks. Moreover, exchanging the scope is also impossible since each scope is paired with an access token. Although it is an inspirational work, a viable solution must make use of dynamic tokens or passwords. Later, in 2015, Singh et al. [28] provided means of security to the MQTT protocol using lightweight elliptic curve cryptography (ECC) to encrypt the broadcast messages. The ECC was preferred to optimize the complexity. First, the publisher prepares an access list to send to the broker and demands an encryption key from the broker. It also adds some information about the user access rights to decrypt the messages and sends them to the broker. If a subscriber holds access permission for a specific message, it also gets the key from the broker and then decrypts the message with this private key. However, no information was given regarding how the key would have been generated. Furthermore, the publisher sends the access tree (a tree-formed data structure representing the access policy) to the broker and gets the key from the broker over a non-secure channel, so that an attacker may easily reach this information by eavesdropping.

In a home automation setup proposed by Upadhyay et al. [29], an access control list (ACL) plugin is added on top of the MQTT protocol to prevent access to sensor data. An ACL is a list file that includes usernames, encrypted passwords, and topic permissions in their model. This approach comes with two major consequences. First, in a dynamic and crowded ecosystem, the addition or removal of devices would be typical and continuous. This behavior naturally causes the addition or removal of new usernames, passwords, and access rights to the list. Hence, the list has to be updated and re-shared repeatedly between the devices, which shall cause an undeniable overhead. Second, this approach only provides authorization but does not enforce any specific authentication control. An MQTT access control system was proposed by Niruntasukrat et al. [30]. It is based on OAuth 1.0 authorization protocol to avoid exhaustive computations. In addition, one more authentication factor was implemented to disseminate a piece of unique credential information over an HTTPS connection to secure the tokens. However, the security issues they addressed are only a portion of the possible risks, as they argued as well. Although providing authorization, the system lacks confidentiality aspects regarding the shared information. It is vulnerable to eavesdropping as well as replay, man-in-the-middle, and denial-of-service attacks.

Zamfir et al. [31] have made a comparative analysis of the security aspects of MQTT and CoAP protocols based on the pre-shared key approach and the use of raw public keys. The pre-shared key approach relies on symmetric encryption and the use of TLS between both ends of the communication. It can be used simply with CoAP but is not natively supported in MQTT. Such uses of TLS increase computational costs, too. The same limitations also apply to the raw public key method. Both protocols support certificates, but they require devastating computational power (and energy). However, in our work, payload encryption is made by AES. Yet, there is no extra computation or use of non-secure channels for key generation and distribution. Rajan et al. [32] proposed a mechanism where the broker uses a secure user profile-based data privacy by hierarchical inner product encryption (HIPE) for data subscription. So, they managed to allow access only to clients who are authorized in real-time video sharing. The study concludes that HIPE encryption and decryption lead to some delays in data transmissions. In addition, role-based access control that requires a tree-like hierarchy is not usually suitable for IoT scenarios.

The RES-256 algorithm introduced by Nagarajan et al. [33] offers an access control mechanism to enhance the security of healthcare systems, based on the so-called Internet of medical things, including advanced encryption mechanisms (e.g., RC6), secure key exchange protocols (e.g., ECDSA) and SHA256. Using RC6 for encryption, ECDSA for key exchange, and SHA256 for data integrity can contribute to securing sensitive data and ensuring the integrity of healthcare information as they are well-established cryptographic algorithms. However, the proposed mechanism does not mitigate the vulnerabilities associated with weak or short passwords due to relying on SHA256. Alshammari [34] proposed another method in the health domain, where the pre-shared public key is distributed to clients by the broker and the username, and the passwords are verified from the database by the broker. Fathy et al. [35] considered an IoT scenario centered on agricultural irrigation with sensors and actuator devices, where the authentication between clients and brokers needs to be ensured. They provided confidentiality solely between clients by utilizing the expeditious cipher (X-cipher) without emphasizing access authorization. The study also compared the runtime memory usage performance of the X-cipher, AES, and PRESENT encryption algorithms.

Shilpa et al. [36] proposed the use of secure reliable message communication (SEC-RMC) protocol along with Mosquitto to perform MQTT data transmission more securely. In the study, a public–private key exchange is made between Mosquitto and clients while it is encrypted via AES for the confidentiality of the data. The broker only verifies the topic information with the database and then shares it with the subscriber. Unlike this scheme, MARAS provides the right to access the topic and the client’s permission to publish or subscribe via OAuth 2.0 protocol. In relation to that, [37] compared the runtime performances of lightweight encryption algorithms in terms of encryption and decryption operations on MQTT payloads. The study evaluates AES-128 Galois/counter mode (GCM), GIFT-combined feedback (COFB), Romulus N1, and Tiny JAMBU. While payload encryption provides confidentiality for the data transfer, it does not address other crucial aspects of secure communication, including authentication, integrity, and protection against attacks like replay and impersonation. Relying solely on payload encryption is insufficient for comprehensive security in MQTT communication. MARAS approaches security holistically and addresses all aspects of secure communication to mitigate potential risks and vulnerabilities effectively.

When asymmetric encryption is preferred, ECC stands as another powerful alternative. Ref. [38] describes implementing a public-key encryption system based on ECC to secure data in an MQTT-based energy management system. Since ECC uses elliptic curves over finite fields to provide strong encryption with relatively smaller key sizes compared to other encryption algorithms, for our future work it may be preferred for resource-constrained devices like Raspberry Pi in IoT applications. Patel et al. [39] also advocated the use of ECC. They proposed a new authentication approach for IoT applications, namely, a two-factor level-dependent authentication for generic IoT (LDA-2IoT), where they utilized elliptic curve cryptography. The proposed approach allows users at a specific level within the hierarchy to access sensor devices deployed either below or at the same hierarchical level. The study conducted a validation of the LDA algorithm within a communication environment based on MQTT over TLS as its secure channel, involving users, gateways, and sensor devices. Using ECC for encryption, Saqib et al. [40] introduced a framework to address significant security concerns in IoT-based networks. Their approach combines elliptical curve cryptography and hash chains to establish a signature-based 3-factor authentication system tailored for IoT environments. Like our proposal, MARAS uses a dynamic session key for mutual authentication to prevent known session key attacks. Payload encryption and access authorization play a crucial role in ensuring the security of their MQTT communications. While the study may not have specifically highlighted these aspects, it is imperative to address payload encryption and access authorization when implementing a secure MQTT protocol.

A 2017 study by Katsikeas et al. [41] applied payload encryption using different AES modes to the data carried by the MQTT protocol. They have concluded that the AES-CBC is the best choice for constrained IoT devices. The study provides a solid knowledge base regarding AES-based encryption schemes (though it does not cover the AES-CFB mode). However, the research merely focuses on confidentiality and ignores other security aspects; yet, there is no explanation regarding the preferred key generation and distribution mechanisms. Without employing any authentication scheme, a malicious subscriber may capture all published information on any topic. Moreover, a malicious publisher can continuously flood spam messages and may eventually cause a DoS attack. Bhawiyuga et al. [42] have used a JavaScript object notation (JSON) web token (JWT) authentication server to authenticate the devices in the MQTT network. This was carried out via a direct token exchange between the clients and the broker; the broker then validates the tokens by sending them to the authentication server. However, although mentioned superficially, their work does not elucidate how a legitimate client device can maintain (or re-establish) its connection after its token is expired. Apparently, both the broker and the authentication server reject the connection requests in such a case. More importantly, while the token is a static one involving a hash algorithm to be protected against integrity attacks, it can still be obtained by eavesdropping. Hence, the token can be reused by malicious nodes repeatedly with a replay attack until it expires. To mitigate such vulnerabilities, a two-step authentication scheme involving a dynamic password mechanism must be implemented (as we did in our study).

Bashir et al. [43] recommended using a trusted third-party server to handle key generation and distribution on MQTT. The server generates unique client IDs and distributes them to the clients. Thus, they are used as a pre-shared key for the encrypted transmission of a second key. The payloads of the clients are then encrypted with this second key. In their scenario, the initial distribution of the IDs will require a TLS connection unless they are sent as cleartext (which is worse). Further, this solution does not prevent replay attacks and flooding of vast messages by malicious nodes. So, attackers may easily overload the message traffic because the broker does not have a suitable device authentication mechanism. In their 2018 study, Wardana et al. [44] presented a prototype of an access control mechanism for IoT devices, which is based on the use of tokens along with the Redis authorization server. In addition, a secure socket layer (SSL) certificate was issued to provide data integrity and confidentiality between the cloud and the MQTT broker(s). The proposed mechanism is very concrete to its extent; nevertheless, it does not include a procedure for broker authentication (by the clients). Another novel security solution for MQTT was suggested by Calabretta et al. [45]. They offered a new approach for the MQTT protocol, based on the augmented password-authenticated key agreement (AugPAKE) algorithm over the ActiveMQ middleware. They also proposed a way to generate authorization tokens for each topic to provide topic-specific access control. Their study supersedes a similar prior work also based on the use of AugPAKE without TLS, published by Shin et al. [46], which lacks authorization features. Despite being broader, [45] ignores the authentication of the broker(s) by the clients, which is critical.

A paper written by Bali et al. [47] points out that a ciphertext-only attacker may also be able to listen to the traffic and decrypt the password right after the broker authenticates the first publisher and subscriber. They proposed a chaotic lightweight algorithm as a precaution against the mentioned ciphertext-only attacks. The algorithm ensures that the key is updated periodically by both the broker and the client(s). This steady work has two minor flaws: first, it uses TLS for the initial authentication; second, it stores a list of topics and authorized clients on the broker. Sundarrajan et al. [48] used advanced multiple encryption systems (AMES) based on the Hardy Wall encryption, requiring less bandwidth and minimal memory while utilizing the MQTT protocol. This is proposed to ensure the secure communication of messages without any packet loss. IoT device IDs and user IDs are also to be encrypted by the Hardy Wall encryption to provide authentication to some extent. However, their model presupposes an unjustifiable trust between the nodes. The paper also lacks information regarding security vulnerabilities, related attacks, and their potential effects, which are vital in such works. Public key cryptography is already a working solution to the authentication problem in MQTT, but key distribution is a major issue in this case. Aknın et al. [49] proposed an architecture that integrates blockchain and smart contracts into an MQTT setup to ensure data immutability and to automate authentication, publishing, and subscribing processes. In their architecture, MQTT protocol authentication relies on the use of one-time passwords (OTPs). To securely transmit the keys required for OTP generation and message encryption, TLS is utilized during the initial phase.

Sanjuan et al. [50], like [39], have proposed using smart cards to distribute keys to clients and brokers. They provided mutual authentication and payload encryption. Although reliable against most remote attacks, their method is vulnerable to physical attacks, like simply capturing the nodes and the smart cards. On the other hand, Amoretti et al. [51] have utilized a cloud server and a token-based authentication scheme. They also added support for multiple brokers via broker bridging. However, their scheme lacks mutual authentication and payload encryption. The issue of securing the MQTT protocol still remains up-to-date, as Blazy et al. in 2021 [52] proposed a comprehensive framework for securing the protocol beyond its basic encryption scheme. Their authentication mechanism relied on attribute-based signatures. While this framework could work well for many IoT systems, it might be too heavy for a network populated with lightweight sensors due to the quasi-complex computation requirements. In [17], the use of multiple MQTT brokers and the authentication of these MQTT brokers were suggested with JSON web tokens. Since the study does not cover security requirements, i.e., client-broker authentication, data confidentiality, and client authorization, it may be exposed to various attacks such as eavesdropping, man-in-the-middle attack, and impersonation attack.

The results of our detailed comparative analysis are provided later in Section 6. According to our literature review, the existing works within this field are either a survey/analysis of the security risks concerning the use of plain MQTT; or a (more conventional) security scheme that is inefficient (i.e., uses SSL/TLS, etc.); or a (more innovational) security scheme that is incomplete (i.e., lacking mutual authentication, vulnerable to eavesdropping, etc.). Thus, having a high-level security approach that is both efficient and complete at the same time is still a paramount concern, which explains why this study is conducted and which problems it resolves.

## 3. Procedures of Security Mechanism

In the recent state-of-the-art, a token and a username–password pair are used to authenticate the IoT devices over the MQTT protocol. There is no standardized or universally accepted security mechanism in the MQTT protocol to provide data integrity, confidentiality, and availability. Furthermore, there has yet been no implementation of a mechanism that allows the clients to verify the broker ID. Three main reasons for the need for a new authentication (and authorization) mechanism for the MQTT protocol can be discussed as follows [26,53].

First, if there is no authentication mechanism, any malicious or compromised device may act as an attacker by either sending (or even flooding) fake messages or accessing all the sensitive data. One main problem in developing a suitable authentication mechanism is to provide means of “uniqueness” to each device in a large network. When the studies in the literature were investigated, the authentication was found to be performed mostly with token-based solutions. However, having a fixed token as a credential allows the identity information to be eavesdropped on easily. To solve this weakness, connecting the devices to an authentication server over a secure channel like HTTPS is evaluated. However, it is found to be impractical due to the use of IoT devices with constrained resources; moreover, there are some reported vulnerabilities [54]. In this study, by applying a second authentication factor using dynamic passwords, a major contribution is achieved in case the static token is stolen. A lightweight one-time password scheme based on an HMAC is used to update the passwords during every connection.

Second, a publisher should make sure that it sends its own messages to the correct broker, and the subscribers must also receive the right messages from the trusted broker. The clients should not be directed to an unauthenticated broker in any case. Mutual authentication has not been offered in the MQTT protocol. In this study, mutual authentication between the clients and the broker is developed by using HOTP with hash chains.

Last but not least, managing devices’ access rights depend on their interest in a topic, and thus messages should not be available to all devices via appropriate access restrictions. In this state, using the security mechanism developed with OAuth 2.0 to provide access control on Mosquitto, the devices are prevented from behaving maliciously.

The proposed security procedure follows three sub-steps: registration, authentication, and authorization, as introduced in detail below.

### 3.1. Registration Step

**Figure 1:**
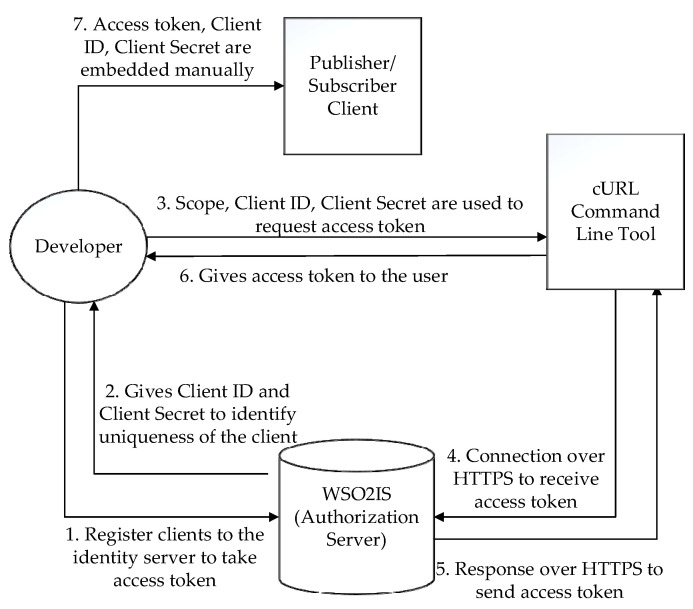
Procedure for the initial registration of the clients to WSO2IS to obtain access tokens.

Initially, a developer manually registers all clients to the WSO2 identity server (WSO2IS) [55]. This procedure is carried out to implement an authorization layer via OAuth 2.0 and to get a unique access token. Remarkably, making that manually may not be the most practical way in some “crowded” scenarios, but certainly, it is the most secure way to do it (without using TLS). WSO2IS creates a client-side secret and the client ID information to obtain an access token. Therefore, the client can achieve unique credentials with an ID and a secret. In order to request an access token for any client, the developer must submit the scope, client ID, and secret information parameters to the WSO2IS via the cURL tool. The registration step is shown in Figure 1 to explain how to access the token in OAuth 2.0 applications. The “developer” in Figure 1 is a responsible person, such as a network owner or an administrator. Flows 2, 4, 5, and 6 are secured through HTTPS.

The second phase of the registration in the IoT application is to obtain the client ID and the client secret for each connected IoT device (i.e., client) from WSO2IS. Then, the access token is obtained via the cURL tool by considering the scope (Figure 1). An example run of the procedure is given in the below paragraphs.

The scope is defined in JSON format for clients as in the example: {“rw”,:“rw”,“Topic”:“\temperature\”}. This means that the client is a publisher who claims both read and write permissions to transmit and receive the “temperature” data to or from the broker. Its base64-encoded version is written as the scope to request an access token from WSO2IS apart from the devices’ client ID and client secret. Our online supplement provides the resulting command (see row 1 of Supplementary Table S2 at srg.cs.deu.edu.tr/publications/2023/maras/—accessed on 2 May 2023).

**Figure 2:**
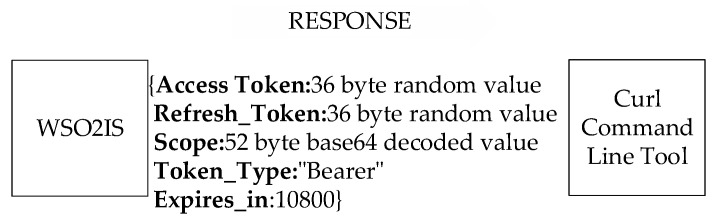
The authorization server’s response to a developer who attempts to register a client node.

The response from the WSO2IS is shown in Figure 2. The scope is parsed into the JSON format to compare with the topic of the received PUBLISH or SUBSCRIBE messages.

The developer manually records the received access token into the corresponding client device, along with the client ID and client secret (as in Figure 1). The access token shall be used to authenticate the client node during its later communications. In this way, an alien device that does not hold a valid access token is prevented from joining the network. The token’s validity period is specified in the “expires_in” field. If the current token is expired, then a new token is issued by the identity server via the “refresh_token” field. However, obtaining a new access token by using that field is not a suitable method for lightweight IoT devices when communicating over an HTTPS connection because this method causes a payload overhead and requires demanding post-processing that may be compelling for IoT devices with limited resources. Therefore, the token lifecycle is kept as long as the application’s lifecycle.

### 3.2. Authentication Step

The username and password are optionally added to the *CONNECT* message when the client connects to the MQTT broker (which is Mosquitto in this study) in order to provide authentication. In the scope of this study, the access token is treated as the username and is used to identify the devices by sending it to the broker via a *CONNECT* message. The broker configuration file is modified with an application that is written in Python programming language. The OAuth 2.0 authorization framework is added as a plugin to this application on the Mosquitto MQTT broker. When a broker receives a *CONNECT* message, it verifies the token on WSO2IS using HTTPS. This design decision relies on the assumption that a broker node can handle an HTTPS connection. If the access token is valid, the broker sends a *CONNACK* message to the client.

**Figure 3:**
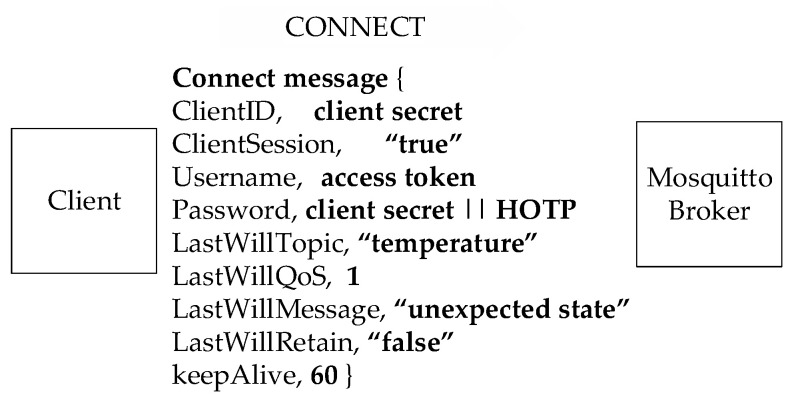
A client’s request to connect a broker via a *CONNECT* message with credentials and scope.

It should be noted that, unlike the HTTPS communication between the broker and the authentication server, a secure channel between the client and the broker has not been considered at all. Further, the token is a static credential and can be stolen occasionally. Therefore, one more authentication factor is added by using a dynamic password that can be generated by concatenating the client secret and a HOTP. The *CONNECT* message is shown in Figure 3, which clarifies the optional use of the username and password.

As stated in Figure 3, the authentication parameters include a username and a password. The username is essentially the access token, which also includes the scope information. The password is an AES encrypted value that contains the client secret (a random number) and the HOTP (another ‘randomized’ number). AES encryption/decryption key is the client ID. It is distributed by the WSO2IS (that runs OAuth 2.0) to the broker(s) through HTTPS, but is manually recorded into the client devices due to security concerns (flow 7 in Figure 1).

The field “ClientID” is necessary in order to identify the client uniquely. It can be a value of up to 65,535 characters as in the MQTT 3.1.1 specifications [12], which is significantly different from the previous version, which allows only 23 characters. The universal unique identifier (UUID), which is 32 characters, the media access control (MAC) address, and the serial number of the devices can also be chosen to provide uniqueness. In this paper, the client secret, which is obtained from WSO2IS, is preferred as the client ID and is 32 characters long. One of the flags in the CONNECT message is “ClientSession”. If it is defined as ‘false’, the broker will keep the client’s message depending on the QoS level. The QoS level can be 0, 1, or 2, respectively. These settings allow storing the message to be transmitted to a client in case of a potentially insecure Internet environment. Usernames and passwords are required to provide authentication and authorization of the clients. Unless hashing or a secure channel is used for username and password, the credentials can be stolen on the go. An eavesdropping attack can be confronted due to transferring the credentials as plaintext from the clients to the broker. The access token is considered a client’s username (a static value) to authenticate the client (by the broker) from the records in WSO2IS via an HTTPS connection.

Furthermore, the password contains a concatenation of the client’s secret and the HOTP in order to provide a dynamic credential. Therefore, the password changes in every CONNECT message (one-time password). Using a one-way hash function makes it more difficult to guess the passwords. The “LastWillMessage” and the “LastWillTopic” (in Figure 3) refer to the last will and testament feature of MQTT. By using them, the clients can be informed about the topic and the message in case of unexpected Internet breaks. The “KeepAlive” field sets the time interval after which the connection between the client and the broker will be sustained without forwarding the message. Whether the client or the broker is reachable is found by the ping messages, PINGREST, or PINGREC.

**Figure 4:**
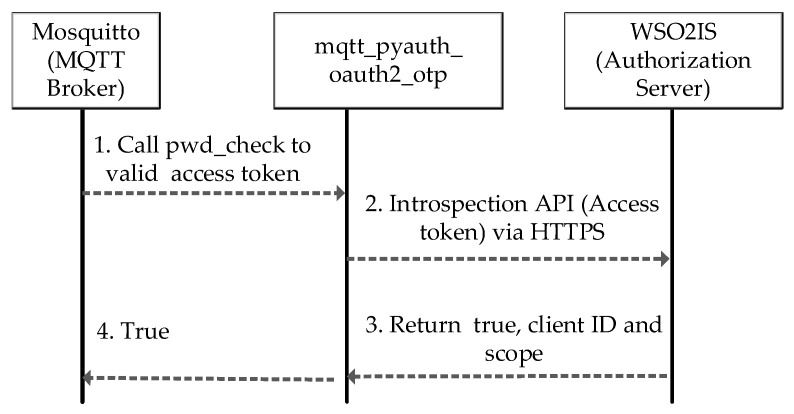
A broker attempts to reach WSO2IS via HTTPS to verify an access token of a client.

The first authentication step takes place by the OAuth 2.0 authorization protocol, and how it is performed is sketched in Figure 4. When an MQTT CONNECT message arrives in the Mosquitto broker, the authentication is performed on WSO2IS by verifying the access token (username). Mosquitto_pyauth [56], a third-party code published on GitHub by Marcin Bahry, provides a Python-based backbone for the Mosquitto broker software. Through personal communication, his code is further developed to extend its functionality to match our security mechanism. Our efforts yielded a novel plugin called MQTT-pyoauth2.0_otp, which runs over Mosquitto to implement the security mechanism. The “pwd_check” function of the MQTT-pyoauth2.0_otp plugin is called to validate the access token among the existing records of WSO2IS. If successful, the client is authenticated by the Mosquitto broker that it is connected to. The MQTT-pyoauth2.0_otp plugin calls the introspection application program interface (API) to verify the access token from the authorization server via the HTTPS protocol. If the client is not registered to the WSO2IS before (or removed), the access token authentication will naturally fail, and the response of the message will indicate that it is an inactive token.

Moreover, if the username and password fields are empty in the CONNECT message, the client’s connection request will be rejected even without calling the introspection API. Thus, Mosquitto will reject an unauthorized client’s request to connect, and a response message will be returned with a CONNACK message to the requesting client. However, if the access token authentication is successful on WSO2IS but it was stolen (due to being static), the second authentication application will be performed. The introspection API returns ‘true’, “scope”, and “client ID” fields in response in case of confirmation of the requesting client’s access token. This client ID can now be used as a key for both AES encryption and HOTP secrets.

**Figure 5:**
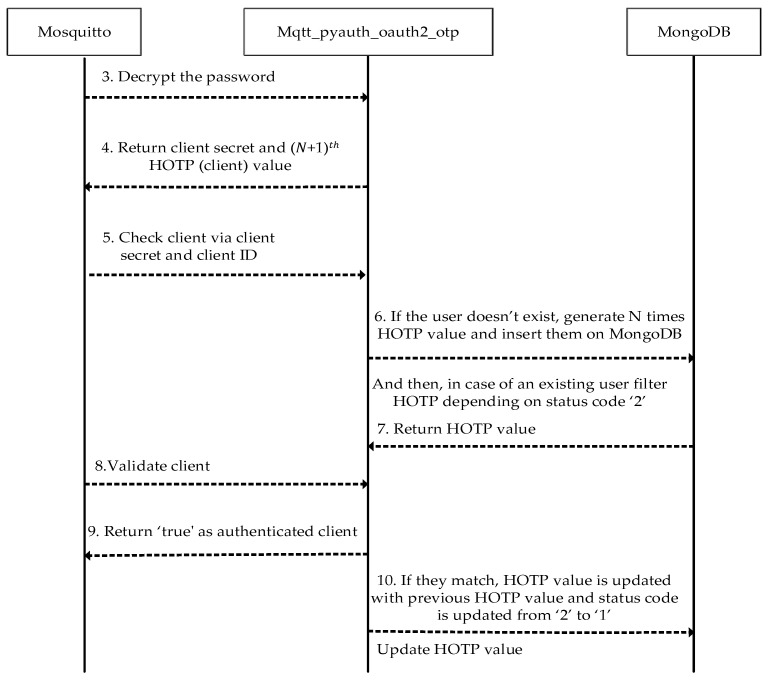
The second step of authentication, during which a broker authenticates a client by checking over MongoDB records.

After the verification of the access token, dynamic passwords should be generated. To maintain that feature, a MongoDB database has been implemented as the backend record-keeping facility in the Mosquitto broker. Dynamic passwords are basically HOTP values that are used to prevent replay attacks. The relevant procedure and its steps are shown in Figure 5. Thanks to the HOTP, a previously issued password shall be invalid after each connection. Thus, a prior connection will be rejected when an attacker has eavesdropped on one password and attempted to connect repeatedly. In addition to this, mutual authentication between a client and a broker is performed by comparing HOTP values. MQTT-pyoauth2.0_otp should generate the HOTP values N times to implement the second authentication step. To proceed, the password is deciphered by Mosquitto using the AES secret key (the client ID) when the active access token is received in the response of the WSO2IS. Thus, the client ID is acquired by the Mosquitto broker. So, as shown earlier in Figure 3, the client ID can securely be known to the Mosquitto broker without sending it from the client to the broker over an insecure channel. This is a crucial functionality. In this way, listening (sniffing) to the client ID or guessing the password and accessing the system by imitating another client is prevented. The diagram of the procedure is given in Figure 5.

The last six digits of the password obtained in the decrypted text represent the (𝑁 + 1)th HOTP value. The remaining sequence of characters is the client’s secret information stored on MongoDB as ‘user ID’ (analogous to Client ID in our JSON format) to provide uniqueness. The client’s secret is searched through MongoDB to find out whether the client has generated HOTP values N times or not. If the client does not exist, N consecutive HOTP values are generated with the MQTT-pyoauth2.0_otp on the Mosquitto and inserted on MongoDB. The synchronization of HOTP between the client and Mosquitto is done with status codes being ‘0’, ‘1’, or ‘2’.

HOTP values with the status code ‘2’ indicate an initiated authentication procedure for the broker and the client. If the status code of a HOTP value that is associated with an existing client secret on MongoDB is ‘2’, the JSON document is updated after the validation of the client so that the status code of the (𝑁 − 1)th HOTP value is changed to ‘2’ from its original value, namely, ‘0’.

**Figure 6:**
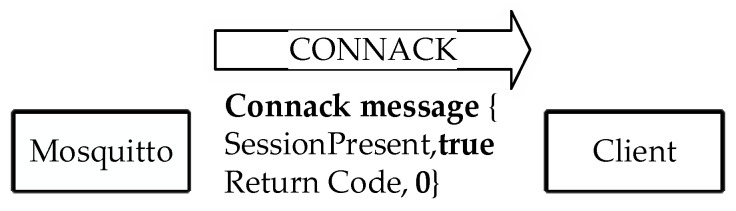
An example *CONNACK* message with return code ‘0’.

After completing the authentication steps, Mosquitto sends a *CONNACK* message to the client. That *CONNACK* message includes return codes ‘0’ to ‘5’. When the client is authenticated successfully, the return code ‘0’ is given in the response message, implying that the connection request is accepted. The remaining codes, namely, ‘1’, ‘2’, ‘3’, ‘4’, and ‘5’, all mean that the connection is rejected, so the connection fails. Two of them, ‘2’ and ‘5’, are directly related to authentication failure. In the *CONNACK* message, in the case of return code ‘2’, a “bad name or password” is returned as the reply, while in the case of code ‘5’, “unauthorized” is returned as the reply. An example *CONNACK* message with return code ‘0’ in our implementation is shown in Figure 6.

**Figure 7:**
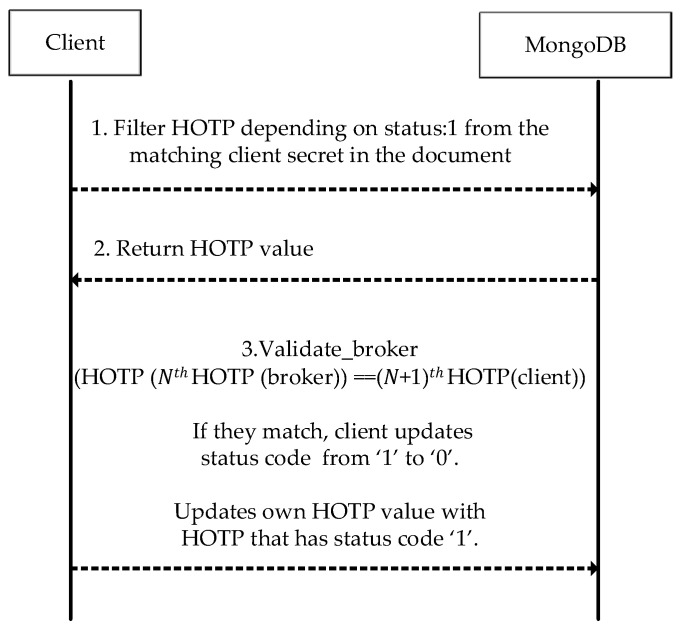
A client’s verification of a corresponding broker; hence, mutual authentication is achieved.

After the client receives a CONNACK message from the Mosquitto broker, in the case of return code ‘0’, mutual authentication between the client and the broker can be achieved using HOTP. This procedure is shown in Figure 7. Before sending a PUBLISH or a SUBSCRIBE message to the broker, the client must first conduct the connection request to the right (and authenticated) broker. When there is no such mutual authentication, the client can easily be redirected to an unauthorized (or worse, malicious) broker. Therefore, this study provides data transmissions between the authorized clients and the authorized brokers. So far, in the existing literature, the security measures have been studied merely regarding client authentication, but neither security issues nor precautions for two-way authentication have been mentioned. The irreversible hash chain function with HOTP is preferred to make the decryption difficult without a known secret.

### 3.3. Authorization Step

**Figure 8:**
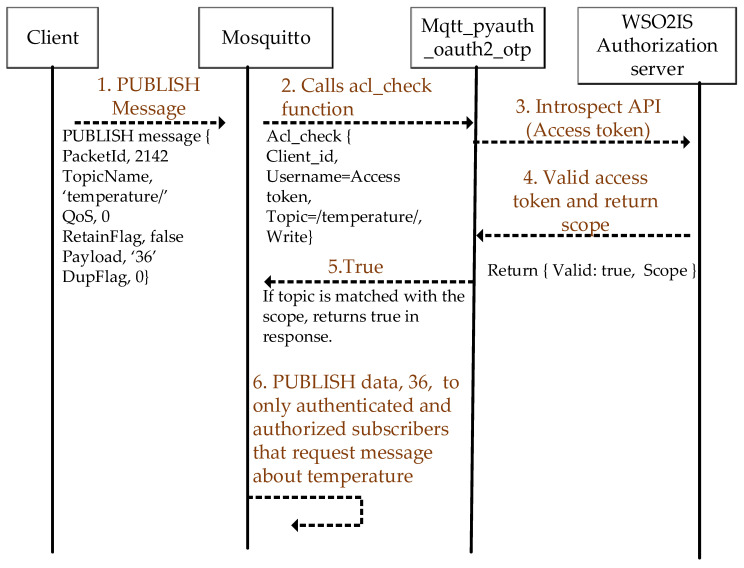
An example *PUBLISH* message and the consequent messaging sequence.

**Figure 9:**
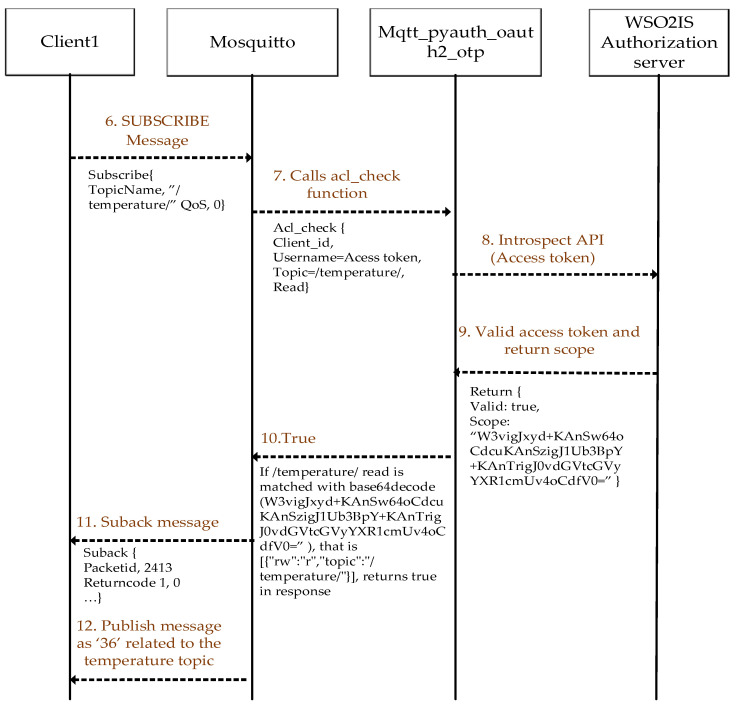
The timeline of a *SUBSCRIBE* message sent from a client to a broker for following a topic.

A client can send messages related to its declared topic(s) of interest to the Mosquitto broker, which indicates that this client is a publisher (of the stated topic). Additionally, the client can request incoming messages from the broker about its topic(s) of interest, which indicates that this client is a subscriber (of the stated topic). Hence, selective data forwarding is essential in MQTT. Access control management is implemented to prevent all the data from being received by all the existing clients. The clients have (and permit) access to data that only belongs to the topics they are interested in. Packet information for both the MQTT PUBLISH and SUBSCRIBE messages for two clients that are a publisher and a subscriber is shown in Figure 8 and Figure 9.

After a client authenticates the broker, the client can send a *PUBLISH* message like in Figure 8. The *PUBLISH* message packet includes a topic name to declare the issuing client’s topic of interest. The topic name is a string, which is indicated with a slash (e.g., “/temperature/”).

The “QoS” field is considered for the reliability of the message transmission in case of an unreliable Internet connection. It may be one of three levels, namely, ‘0’, ‘1’, or ‘2’, depending on the foreseen guarantee of the transmission of the message. When the level ‘0’ is considered, the protocol transmits the message at most once and thus, does not deal with the potential loss of the message. A message is sent only once and is not further stored on Mosquitto. When the level is set to ‘1’, the message is stored until the client receives the PUBACK message, which will ensure that the message is transmitted at least once. The “PacketID” field is used to match the PUBLISH message packet with the corresponding PUBACK message packet. At level ‘2’ (i.e., the last level), the protocol provides the most reliable transmission by delivering a message exactly once so that it ensures the message is received and acknowledged over four handshake MQTT message packets. After the client transmits the PUBLISH message to the broker, the broker sends the PUBREC message to the client, confirming that it has received the PUBLISH message. If the client does not receive the PUBREC message, it sends the PUBLISH message repeatedly until it receives the PUBREC message with the “DupFlag” (i.e., duplicate flag) field.

Following the reception of the *PUBREC* message, the client sends the *PUBREL* message with the packet ID, and the broker sends the *PUBCOM* message to the client as a reply, ensuring that the message is available again. The use of the “DupFlag” field is necessary in the case of both QoS ‘1’ and QoS ‘2’ levels. Like the “DupFlag”, the packet ID is also important in situations other than the QoS level ‘0’ as it defines the uniqueness of the messages. The “Payload” field specifies the message content. The QoS level ‘2’ causes higher overheads and is rarely used in lightweight networks.

When the Mosquitto broker receives a *PUBLISH* message from a client, it calls the “acl_check” function from mqtt_pyauth_oauth2_otp application to decide whether the client is authorized or not to send such messages about the specified topic. The application calls the introspection API with the corresponding access token via HTTPS from WSO2IS to validate the client and obtain its scope information. If the access token is valid, the scope information is returned as “*base64decode*”, and then the application parses the scope and compares the received topics from the client with the parsed scope information. If the client’s topic information and the access permission match the scope information owned by the access token, then the client’s authorization is verified. The clients may act as a publisher or a subscriber. In any case, they do not transmit their data to each other directly. All the communication is taken place over the Mosquitto broker(s).

When the broker receives a *SUBSCRIBE* message from a client, the procedure steps in Figure 9 are followed to decide whether the client is authorized or not to receive messages on the specified topics. After verifying the client’s access rights in correspondence with its topics, such as data write and read, the data transmission can successfully be completed. Additionally, the *UNSUBSCRIBE* message is used to remove the request of a subscriber from Mosquitto. It includes the subscriber’s topics and packet IDs.

If the read and write access permissions for a stated topic do not match the scope information in the valid access token, the client is declared unauthorized. Hence, no message will be sent or received (to/from that client). In the case of an invalid access token, the response is returned as an unauthorized client without calling the “acl_check” function.

## 4. Implementation and Validation

An example case of the proposed scheme is implemented for functionality validation and performance evaluation purposes. While this section elaborates on the functionality aspects, the performance tests are provided in Section 5.

### 4.1. Preliminaries

Our experiments were performed through emulation of wireless sensor nodes and other networked devices on a personal computer considering the following details:

#### 4.1.1. Evaluation Criteria

Considering the objectives of our work (see the problem statement in Section 1), we have defined three main security features to assess the functionality of our scheme. Accordingly, any MQTT ecosystem that implements MARAS as a security layer must:

- Ensure the uniqueness of every single connected device (i.e., clients, brokers, and the server);
- Allow the Mosquitto brokers to verify every single device (i.e., clients and the server) on its local network, as well as ensure that the clients validate the broker reciprocally as well;
- Merely allow the clients with legitimate access rights to receive and transmit data through the network.

**Figure 10:**
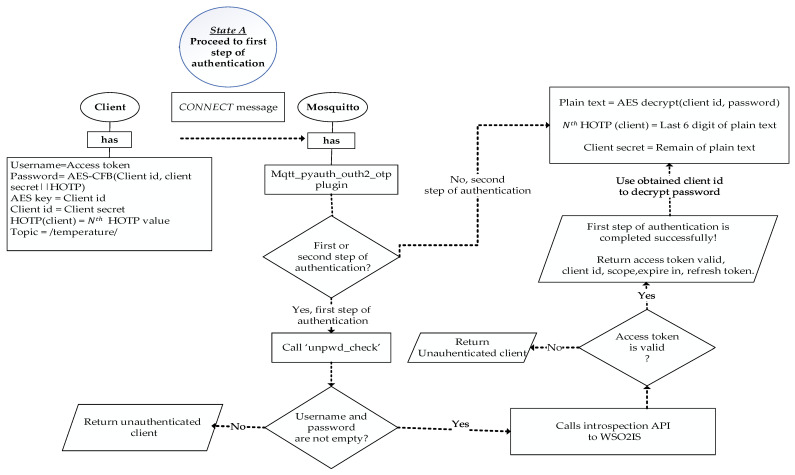
Flowchart of the authentication and authorization mechanisms MARAS implements.

**Figure 11:**
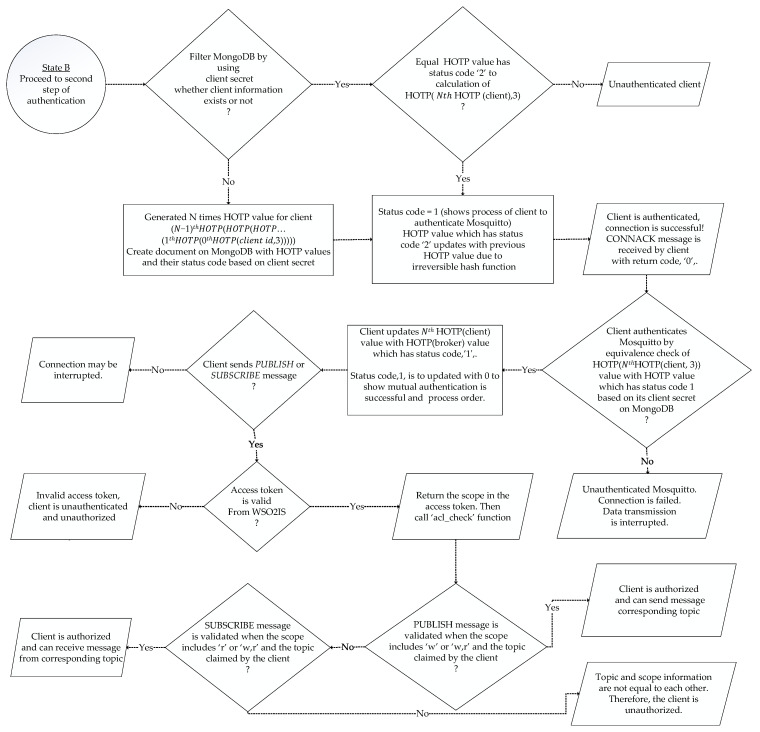
General flowchart of the authentication and authorization mechanisms (second step).

Altogether, our implementation directly follows the flowcharts given in Figure 10 and Figure 11, where the general procedures of the MARAS scheme are illustrated. Therefore, as explained later, our demonstration successfully provided all the above criteria.

#### 4.1.2. System Model, Assumptions, and Configurations

In the proof-of-concept implementation of this work, the following entities were (virtually) created on the same physical computer (equipped with Intel i7 3630QM and running Ubuntu), where the inter-node communications took place within the local host:

- One server that runs the OAuth 2.0 protocol;
- One MQTT broker that implements Mosquitto, an open-source message broker written in C language (mosquitto.org—accessed on 2 May 2023);
- Six clients with both publisher and subscriber roles through the Eclipse Paho Python client library (eclipse.org/paho—accessed on 2 May 2023).

The clients (i.e., sensor/actuator nodes) pose the following capabilities:

- Producing any sort of data that can be transmitted as a byte stream (for sensors), reading such data (for actuators), or even both (for compound devices);
- Sending and receiving data and control messages through the wireless channels;
- Processing power is enough to perform AES encryption and decryption without any considerable lag.
- In addition to the above, the brokers can do the following:
- Forwarding messages within the network depending on the source and destination addresses they contain;
- Dropping messages sent from or destined to unauthorized devices, per permissions in the server.

The OAuth 2.0 protocol is preferred to issue authorization privileges without revealing the usernames and passwords of the clients. This helps to protect user credentials from being reused without consent (or even awareness) of the clients (and human users) by malicious applications and/or attackers [57]. For the sake of reproducibility, we present the rest of the test settings in the online Supplementary Materials (see Supplementary Table S1 at srg.cs.deu.edu.tr/publications/2023/maras/—accessed on 2 May 2023).

#### 4.1.3. Test Scenario

In order to validate the system’s ability to satisfy the given objectives and functionalities introduced in the previous section, a test implementation was made. Per the test case, six clients (IDs from 1 to 6) with different privileges have been defined in the local network. The topic information that the clients are interested in and the read/write access permissions (i.e., scopes) are shown in Table 2. The scope information of these clients is also specified on the server. Then, the client ID and client secret information are obtained for each client. Therefore, it is shown that the client ID and client secret are unique parameters for each client. The information in Table 2 is used to obtain access tokens via the cURL command given in the online Supplementary Materials (see row 1 of Supplementary Table S2 at srg.cs.deu.edu.tr/publications/2023/maras/—accessed on 2 May 2023).

After obtaining an access token via the cURL command, the client ID, client secret, access token, topic, HOTP value, port number, and the broker’s address are manually recorded in the client. The information base of Client 1 is shown in Table 3 as an example.

When Mosquitto receives CONNECT messages from the clients, the first authentication step is run by verifying its username on WSO2IS via a secure channel created with an HTTPS connection. The contents of the CONNECT message packet are not given as they are not human-readable (base64-encoded or AES-encrypted).

**Figure 12:**
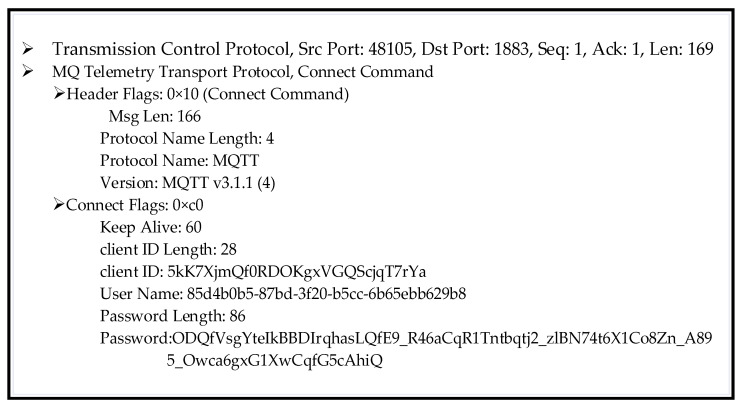
A *CONNECT* message containing credentials sent from client 2 to a broker.

Wireshark is used to interpret and analyze the collected data over the MQTT protocol as a network traffic sniffing tool. The raw contents of the sniffed CONNECT message packet of Client 2 are shown in Figure 12.

The MQTT packet includes a two-byte fixed header, in which the first four bits include the MQTT message types and the last four bits include the control flags. The header flag is defined as ‘0 × 10’, which means that the first four bits are ‘0001’. This setting points out a CONNECT message type, and the last four bits are ‘0000’, which indicates the control flag. There are 14 message types defined by four bits in the data packets. Additionally, the control flag includes a duplicate delivery (DUP) flag for a PUBLISH control packet, the quality of services (QoS) indicator, and the retain mode flag. These flags are defined, respectively, by one, two, and one bit as in ‘0000’. The variable-length header contains a control flag that is not mandatory in all message types. A CONNECT message data packet contains the control, length, protocol name, protocol level, connect flags, the “keep-alive” field, and the payload. The payload includes the client ID (28 bytes), the username (36 bytes), the password (86 bytes), will topic (1 bit), and message (1 bit) flags. The control flag is defined as ‘0×c0’ in the above example. That means the username and password flags are set to ‘1’, whereas the remaining flags of retain (1 bit), QoS (2 bits), clean (1 bit), will (1 bit), and reserved (1 bit) are all set to ‘0’ (i.e., ‘1100 0000’). The total packet length is equal to 169 bytes. That length is the sum of the header flags (1 decimal) + protocol name and version (5 decimals) + connect flags (12 decimals) + keep-alive (1 decimal) + client ID (28 decimals) + username (36 decimals) + password (86 decimals).

The username is essentially validated from WSO2IS via HTTPS. If it is found to be correct, the client ID information is then returned to the Mosquitto. An example response is provided in the online Supplementary Materials (see row 2 of Supplementary Table S2 at srg.cs.deu.edu.tr/publications/2023/maras/—accessed on 2 May 2023).

Otherwise, if the username is not valid, WSO2IS returns a different message reporting the failed attempt regarding the username validation (see row 3 of Supplementary Table S2 at srg.cs.deu.edu.tr/publications/2023/maras/—accessed on 2 May 2023). If the access token is false, the Mosquitto returns to the client as it is an unauthenticated client with the MQTT CONNACK message code as ‘5’. Another possibility is that when the username is empty, Mosquitto responds with a CONNACK message as a bad username or password, and the connection is rejected.

### 4.2. Mutual Authentication

The mutual authentication of the parties involves the authentication of any client by the broker, and the authentication of the broker by all clients, respectively. As the initial step of the authentication measures, the connection requests from recently deployed clients that were not previously registered to the local network are rejected due to the absence of their corresponding access tokens. However, since the connections are established through insecure channels, it is possible that a static access token can be sniffed by a malicious device so that an attacker can connect to the Mosquitto broker. Yet, the second authentication step enforces using a dynamic password that is changing after each connection to prevent an attacker from connecting to the server with a previously sniffed static access token. Likewise, the attacker is prevented from proceeding with a replay attack due to the enforced use of replaced dynamic passwords during data transmission to the Mosquitto broker. To achieve that, HOTP is concatenated with the client secret to build a unique password on each connection. HOTP also makes it difficult to guess an issued password. Moreover, the confidentiality of this password is ensured by AES encryption. Since the clients are not allowed to communicate with each other directly, all communication occurs through the broker(s).

Therefore, the AES key should only be known to the Mosquitto broker. Instead of transmitting the key from an insecure channel to the Mosquitto broker, the client ID is preferred as the AES key that is already known by both the corresponding client and the Mosquitto broker. The password is decrypted via client ID by padding ‘0’ to generate a 32-byte AES key. Later on, the client secret and (*N* + 1)th HOTP (client) values are obtained as in Table 4.

The broker acts as a generator for generating and verifying a HOTP value, whereas the client stores only the final HOTP value. Afterward, the HOTP value is updated according to the verification result on MongoDB.

Within the second authentication step, the first thing to search on MongoDB is whether the broker generated the HOTP value depending on the corresponding client’s secret or not. If not, the broker creates HOTP values N times and records them on MongoDB. In our tests, the sampling is done by assuming that N is 6. In order to provide synchronization between the client and the Mosquitto broker, status codes such as ‘0’, ‘1’, and ‘2’ are kept along with each HOTP value depending on the client’s secret. These status codes represent a sequence of a process for a client and the broker to compare their HOTP values and to calculate a new HOTP value when necessary. For example, for an existing user, say Client 1, the user data document stored on MongoDB (containing a list of recently generated HOTP values) would look like the entries given in Table 4 [21].

Seven consecutive HOTP values are generated using the client ID and the number ‘3’ as the secret, respectively. The obtained HOTP values are given in the online Supplementary Materials (see Supplementary Table S3 at srg.cs.deu.edu.tr/publications/2023/maras/—accessed on 2 May 2023).

**Figure 13:**
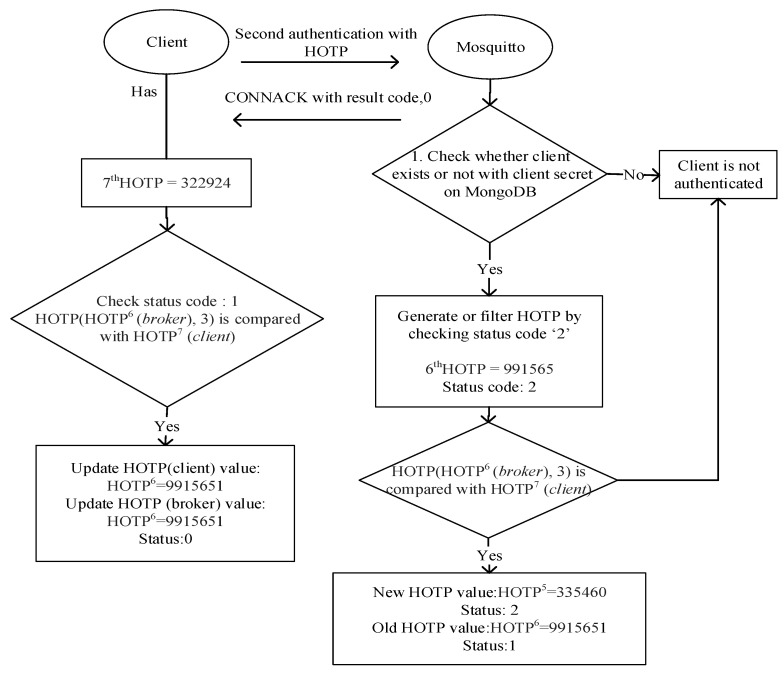
Flowchart showing how the second step of authentication is executed using HOTPs.

The 7th HOTP value is calculated by updating the secret using the broker’s HOTP value, which has the status code ‘2’ (see Supplementary Table S4 at srg.cs.deu.edu.tr/publications/2023/maras/—accessed on 2 May 2023), and then this value is compared with the client’s HOTP value. If and only if the values are equal, the document based on the client’s secret is updated as in the bottom part of Figure 13.

**Figure 14:**
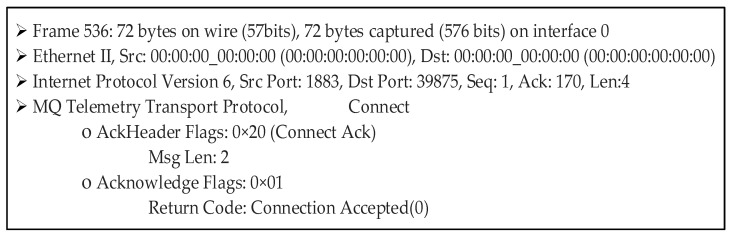
A *CONNACK* message sent from a broker to client 2 showing the client is authenticated.

A *CONNACK* message is sent to the client when the client is authenticated by comparing the calculated HOTP value (which has status code ‘2’) and the HOTP value (client) on Mosquitto. At the same time, the broker’s HOTP value is updated with the previous HOTP value to make it impossible to guess the HOTP value owing to the use of an irreversible hash function. The contents of the *CONNACK* message data packet are given in Figure 14 (obtained via Wireshark).

As interpreted in the given dump records, the authentication is successful and the *CONNACK* message is received with the return code ‘0’, which means that the connection request is approved. Before sending or requesting a message on the topic of which the client is concerned, the client must ensure that it is communicating with the genuine broker. Therefore, mutual authentication is implemented using the HOTP value within a hash chain. A client device authenticates the Mosquitto broker by comparing the client’s own calculated HOTP (generated using the client ID and the secret, namely, the number ‘3’) to the broker’s HOTP value, which has the status code ‘1’. If they are found to be identical, the client updates its HOTP value with the HOTP value, whose status code is ‘1’. This process [23] is shown in Figure 13. Theoretical problems of possible interruptions in the connection are detected using the status codes that virtually ensure the synchronization of the HOTP values.

In case the *CONNACK* message with result code ‘0’ is received, the client authenticates the broker, as illustrated in Figure 13. In case the comparison yields equal values, the client’s HOTP value and the document based on the client’s secret are further updated as in Figure 13.

### 4.3. Client Authorization

**Figure 15:**
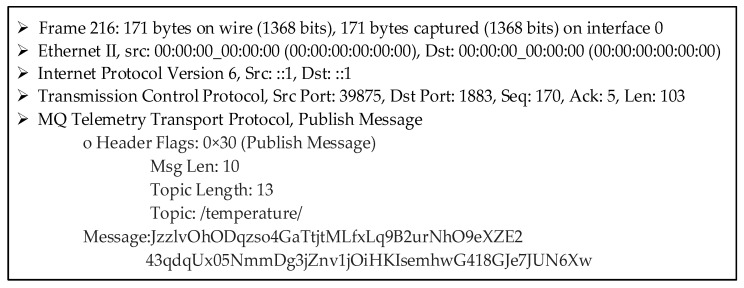
A *PUBLISH* message sent from client 1 to a broker after successful authorization is maintained.

Consider Client 1 publishes a message (i.e., string) containing the characters ’36 °C’ (an example temperature value) after the successful mutual authentication. The confidentiality of the message is provided by AES encryption. This published message data packet is broken down using Wireshark in Figure 15.

Assuming the mutual authentication of each client is successful, each client should only be allowed to access the messages based on its own read/write access permission. Otherwise, (possibly irrelevant) data can be exchanged with all connected clients, even the not-interested ones. In that case, each unauthorized client will significantly inflate the message traffic. In this study, the access control mechanism is provided by using an OAuth 2.0 authorization server that gives scope information with a valid access token.

When a Mosquitto broker receives a PUBLISH message, first, the scope information is obtained from WSO2IS if there is a valid access token. The scope is returned in JSON format, and then the parsed scope is compared to the topic information (which is ‘temperature’ for Client 1). If the compared values are identical, Client 1 is authorized and is allowed to send its message. In Table 5, all clients’ authorities are observed when the authorized Client 1 sends a PUBLISH message to the broker regarding the topic ‘temperature’. The message contents are given in the online Supplementary Materials (see row 4 of Supplementary Table S2 at srg.cs.deu.edu.tr/publications/2023/maras/—accessed on 2 May 2023).

As shown in Table 5, Client 7 does not have an access token because it is not registered on the WSO2IS authorization server. So, Client 7 cannot gain authority for any topic, and the response returns to the unauthenticated client regardless of calling the ‘pwd_check’ function. The remaining clients are authenticated owing to posing valid access tokens and valid HOTP values. However, both Client 5 and Client 6 have ‘read’ permissions for different topics; the requested topic does not match their authority, so ACL is denied, whereas Client 3 *holds* written permission for a different topic (i.e., ‘humidity’), the ACL is rejected since the topics do not match. Both scope and topic permissions of Client 1, Client 2, and Client 4 do match; hence, ACL is successful. A disadvantage of the access control mechanism that we recommended is *that* it cannot be updated after the scope is once defined for each access token. We plan to solve this problem by expanding the access control model we developed using mobile applications as a future study.

## 5. Performance Evaluations

This section includes a brief complexity analysis and provides a detailed experimental evaluation.

### 5.1. Complexity Discussion

The time complexity of the procedure for registering the clients to WSO2IS to obtain access tokens, as visualized in Figure 1, is found as O(N), where N is the number of clients in the network, because the registration must be repeated for each client device. The complexity of connecting a single Mosquitto broker to WSO2IS via HTTPS to verify an access token, as given in Figure 4, is O(1); yet, this process can be executed in a parallel fashion if there is more than one broker. The second step of the authentication illustrated in Figure 5, where a broker authenticates a client over MongoDB records requires N calculations for HOTP (if the client was not registered in MongoDB) and this must be repeated for each of N clients; therefore, O(N^2^). Introduced in Figure 7, a client’s efforts on verifying the corresponding broker (to secure mutual authentication) again require N calculations of HOTP. Although this step is executed for each client, the execution is parallel on each client device; hence, O(N) complexity is achieved. So, the time complexity for a *PUBLISH* (Figure 8) or *SUBSCRIBE* (Figure 9) message remains at O(N^2^) as it will be the dominant term in the sum of all partial complexity classes given above. Encryptions and decryptions with AES on both clients and brokers have O(1) time complexity, and thus, can be neglected. On the other hand, the message complexity for both message types turns out to be O(M), where M is the message to be sent. Because each *PUBLISH* message requires 6 messages to be sent within the network, likewise, each *SUBSCRIBE* message requires 7 messages to be sent. Since the increase is a constant factor, the complexity class is not affected.

### 5.2. Runtime Performance

The runtime performance of the proposed method is measured during a test run made with seven clients, a Mosquitto broker, and a WSO2IS authentication server, acting similarly to the scenario described in Section 4. While the tests focus more on network performance, it is also possible to estimate the clients’ energy consumption. The clients’ roles are again defined per Table 5, except Client 7 (which is now a publisher of ‘temperature’). So, four clients (1, 2, 3, 7) have been set as publishers (of some temperature, humidity, and pressure data), and three clients (4, 5, 6) have been set as subscribers of this information. Some comparisons are made between the case of secure communication (as described in this paper) and the case of non-secure communication to objectify the cost of applying the proposed method, namely, MARAS. Wireshark is used to track the network traffic throughout the communication of the parties. The statistics are measured via Wireshark, too.

Following the initialization of the system, Client 1, as a publisher, sends a burst of 100 periodic messages containing the temperature data ‘36’ and stops. Three minutes later, Client 1 and Client 2 together publish 100 messages (200 in total) on ‘temperature’. After another three minutes, Client 1, Client 2, and Client 3 all publish 100 messages (300 in total) on their topics. When the third three-minute waiting period is over, lastly, all publishers (i.e., Client 1, Client 2, Client 3, and Client 7) publish 100 messages (400 in total) on their topics. Thus, a total of 1000 messages are sent in 4 consecutive burst sequences that make a total of 9 min. The messages are sent both as encrypted and in plaintext so that the performance is observed by calculating the packet overheads and the round-trip time (RTT).

**Figure 16:**
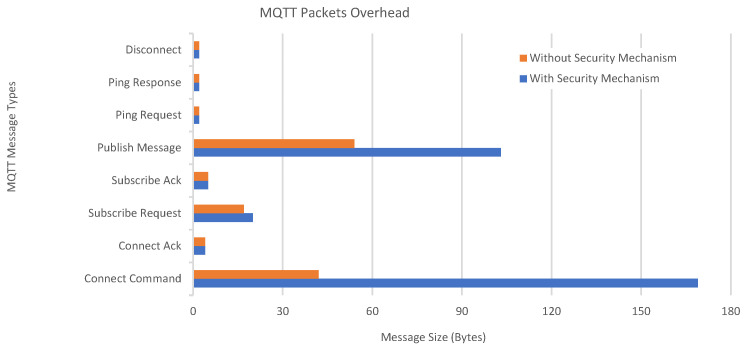
The traffic overhead generated by MARAS compared to non-secure MQTT communication per various message types.

Figure 16 compares caused traffic overheads between the equivalent communication sessions executed with and without the proposed security method per different MQTT message types. The graph unveils an apparent redundancy to *CONNECT* and *PUBLISH* messages. AES encrypted version of a *PUBLISH* message is 103 bytes long, whereas its plaintext counterpart is only 54 bytes (in the case of using 28 bytes for the client ID and 13 bytes for the topic). A *SUBSCRIBE* message is only 3 bytes longer if MARAS is applied. MARAS has 169 bytes-long *CONNECT* messages in a total of 237 bytes-long packets, whereas without MARAS, pure MQTT has 42 bytes-long *CONNECT* messages in a total of 110 bytes-long packets. Yet, other message types yield very similar results.

**Figure 17:**
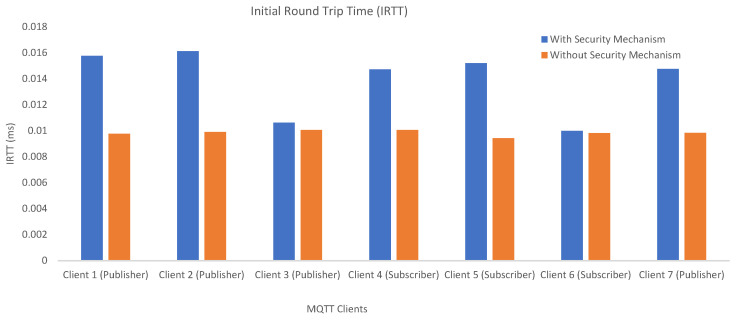
The RTT values at each client compared with and without using MARAS, obtained by sending a *CONNECT* message and receiving a *CONNACK* message in response.

Figure 17 illustrates the RTT values of an arbitrary “initial” CONNECT message for each client. Although somewhat situational, RTT is an indicator of the caused traffic overhead and is used to measure communication latencies in milliseconds. It is found by calculating the time between the transmission of a CONNECT message (sent by the client) and the reception of a corresponding acknowledgment message, namely, a CONNACK message (sent by the broker). In detail, the process starts whenever a client has to publish a data message to the broker. Later, the broker decrypts the received CONNECT message, interprets it, and sends a CONNACK message back. Whenever the client receives the acknowledgment, the RTT is calculated.

From Figure 17, the RTT values of clients 1, 2, 4, 5, and 7 become significantly higher when MARAS is applied, whereas it has a negligible effect for clients 3 and 6. It is because clients 3 and 6 lack the required scope information that they cannot make use of secure communication. Hence, without secure communication, their packets stay smaller and float faster. Therefore, the security mechanism does not degrade any non-secure communication that takes part in different slices of a network.

Considering the test case mentioned, the traffic over the wireless channel can be analyzed in four stages per four message bursts, as explained below:

- In the first three minutes: only one publisher sends 100 messages while three subscribers listen;
- Between the third and sixth minutes (excluding 6:00): two publishers send 100 messages each while three subscribers listen;
- Between the sixth and ninth minutes (excluding 9:00): three publishers send 100 messages each while three subscribers listen;
- At the ninth minute: four publishers send 100 messages each, while three subscribers listen.

**Figure 18:**
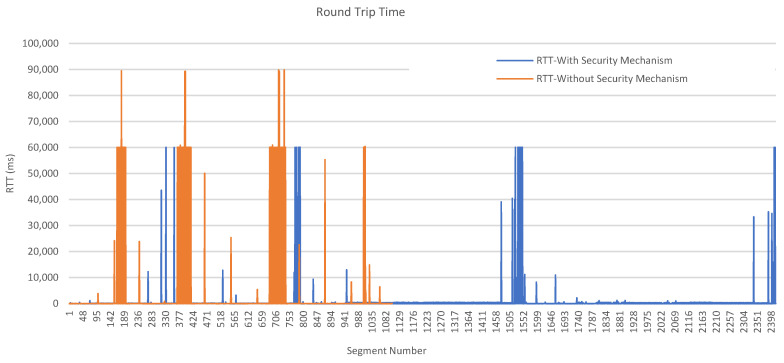
The RTT values for all TCP segments sent by a client, compared with and without MARAS; each segment contains 1000 *PUBLISH* messages sent through the channel.

In Figure 18, the RTT values are shown for every single TCP segment that constitutes a total of 1000 PUBLISH messages sent by all four publishers. Unlike the previous experiment, the measurements here are provided by Wireshark’s TCP analyzer and do not consider the PUBACK messages but the TCP ACK responses. The segment numbering is continuous so that the numbers do not reset for each message. As described in the scenario, the number of MQTT messages released to the medium increases over time. From Figure 18, this behavior is even more evident in the secure case. In fact, the non-secure communication requires only 1417 packets, whereas the security mechanism requires 2414 packets for the same data to be transferred (but securely). Thus, there is a clear tradeoff.

**Figure 19:**
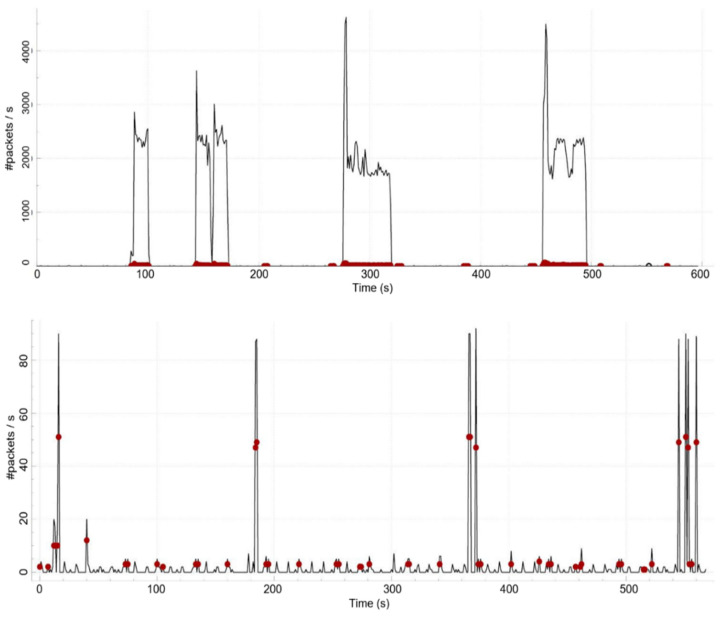
Volume of data traffic generated by bursts of *PUBLISH* messages sent by a client, compared with (**bottom**) and without (**top**) using MARAS.

Figure 19 shows the data traffic on the wireless channel during the mentioned above test scenario for both secure and non-secure communication. The lines show all packets in the TCP traffic, whereas the red dots represent the MQTT packets (on TCP port 1883). Additionally, green squares would represent a packet loss, but no loss has been recorded. Although the bursts of PUBLISH messages are easily observable in both cases, the traffic volume is significantly higher when the security mechanism is applied. Nevertheless, the congestion is far from being disruptive for any modern IoT or WSN deployment.

### 5.3. Comparative Analysis

Moving forward with the runtime performance of MARAS obtained in our tests, we provide a brief comparative outlook referring to similar schemes from the literature. Information provided in this part is not a comprehensive analysis as the test environments are not equivalent, yet it presents an insight into how MARAS compares to alternatives in terms of computation and communication overhead. Our focus is limited to the mutual authentication phase since the registration phase of MARAS is manually performed once by a developer. For this purpose, the computation and communication costs of MARAS are compared to Patel et al. [39] and Saqib et al. [40].

The main goal of designing MARAS is to minimize the processing load on the resource-constrained publisher and subscriber clients while performing computationally intensive operations on the broker(s). The computation cost in MARAS emphasizes the number of cryptographic operations used during mutual authentication and the time required for these operations on the client and broker sides.

Table 6 presents how much time it takes for an Eclipse Paho subscriber/publisher client and for a broker to complete the given operations on our testbed PC running Ubuntu OS with an Intel i7 3630QM processor. In the notation for Table 6 and Table 7, T_E_ is time for symmetric encryption (i.e., AES in MARAS), T_D_ is symmetric decryption that is equivalent to the encryption, T_H_ is time for a one-way hash function, T_HOTP_ is time for calculating HOTP(s), T_ECA_ is ECC point addition, T_ECM_ is ECC point multiplication, and T_AS_ is time for token-based authentication steps performed with introspection API over HTTPS. In our case, the convergence times are identical for clients and brokers as we run them on identical systems. In a real deployment, a broker’s performance can be expected to be slightly (or remarkably) better than the clients.

Table 7, on the other hand, provides a comparative outlook on how many (and which) operations are required on various device types with different methods, as well as how much time is required to complete those operations. According to the results, MARAS performs better than both [39,40]; however, we once again note that the testbeds are not equivalent. MARAS requires significantly more operations than the others on the broker side (as a result of our motivation to offload the heavier operations to the broker); however, the major contribution to the number is the repeated hash function, which is actually a very fast operation. For more information on the compared works, we encourage the readers to read [39,40], while we must note that Table 4 in [39] contains an error regarding the calculated times, which we corrected here in Table 7 as 792 ms.

Table 8 compares communication costs in terms of sent bits in a scenario where there is one publisher client, one subscriber client, and one broker so that a publisher seeks authorization to send some data to the subscriber over the broker. With MARAS, a client sends a broker 28 bytes of client ID + 36 bytes of token (i.e., username) + 86 bytes of password, which sum up to 150 bytes (1200 bits). A broker sends the authentication server data totaling 334 bytes (2672 bits) over TLS; later, it sends an ack to the client totaling 2 bytes (16 bits). While refs. [39,40]’s costs are equivalent, communication with MARAS costs less than both when a public channel is used and more than both when a TLS channel is used.

## 6. Discussion of Results

This part summarizes and discusses the functionality validation introduced in Section 4 and the performance evaluation given in Section 5.

Our proof-of-concept implementation showed that MARAS is an easy workaround for securing two-way MQTT communications between numerous sensor/actuator devices (i.e., clients), intermediary gateways, and servers. It is shown that authorization (of clients) and two-way authentication (of clients and brokers) work flawlessly so that all devices (i.e., clients, brokers, and the server) included in a MARAS-enabled MQTT network environment can ensure the authenticity of every other connected device. MARAS implements dynamic encryption using HOTP for authentication, effectively preventing replay attacks and man-in-the-middle attacks by rendering password reuse invalid in case of interception. Additionally, permission-based authorization (for sending and receiving data) is enforced on sensor/actuator devices by an identity server through the broker(s). By utilizing OAuth 2.0, the IoT application can efficiently manage access control and delegation for a growing number of devices. Each device is authenticated and authorized individually, and its unique ID is used to enforce access restrictions. This granular level of authorization ensures that devices can only access messages and resources that they have been explicitly granted permission to, enhancing the overall security and control of the IoT ecosystem.

We provide our comparative analysis regarding the security features of MARAS and existing schemes in Table 9, where authorization stands for simple access control (e.g., OAuth 1.0, access control list, tree hierarchy, etc.), the role-based access control means advanced node/client authorization by assigned roles (e.g., via OAuth 2.0), client authentication means brokers authenticating clients (e.g., via tokens, user name, and password), HOTP stands for the use of HMAC-based one-time passwords for authentication, mutual authentication means clients and brokers authenticate each other reciprocally (e.g., via HOTP, SSL, nonces, etc.), and confidentiality means payload encryption (e.g., AES). As reflected in Table 9, no previous attempt could collectively provide all the security features that MARAS provides.

Our tests also revealed the effects and costs of applying the proposed security mechanism on top of an MQTT setup. The results show that there is an apparent tradeoff between the communication overhead and the security provided. The actual cost of implementing MARAS (in terms of data overhead) can be found using Table 10, where the three largest message types are compared. The other message types are not only much smaller but also not significantly affected by MARAS; therefore, they can safely be ignored.

From Figure 16 and Table 10, in a long-lasting sensor network scenario where the number of data packages to be disseminated to the network is larger than 1000, the PUBLISH messages will most likely dominate the network’s traffic because the other messages are only sent once (or occasionally). Yet, 1000 is a very modest number for many scenarios as a sensor network may cast millions of messages in its entire lifetime. Therefore, although the cost increase is larger in CONNECT messages, the main cost comes from securing the published data. Hence, MARAS shall double the total traffic overhead compared to the non-secure case. However, assuming there is no multimedia content, the message sizes are still tiny. So, this overhead may be tolerable in most cases if the security of the network is a major concern. Yet, our repeated tests showed that round trip times for a CONNECT message (and its “ack” counterpart) are only delayed by less than a percentile of a millisecond. For a PUBLISH message, the delays again depend on the size and frequency of the published information, but we can safely say that the delay is upper bounded by 163% of the network defaults. Moreover, (from Figure 18), the upper bound of the RTT of a segment (consisting of 1000 PUBLISH messages) does not increase with MARAS. So, the scheme’s degrading effects on the system performance are limited and tolerable.

We have seen that while there is an increased data overhead (up to 2×) and potential latency (up to 1.6×), the overall impact on system performance has remained within acceptable limits for most IoT setups. The additional overhead can be considered a necessary cost for enhanced security, which includes mutual authentication, role-based authorization, and payload encryption. There are also additional tradeoffs when implementing MARAS in IoT applications. These include the impact on energy consumption, scalability, and complexity. The additional processing and communication overhead can increase energy consumption, especially in battery-powered devices. Scalability is also a critical factor to consider in large-scale IoT deployments. The overhead introduced can limit scalability as device configurations must be done exhaustively (e.g., the device registration phase for the identity server), although it can be automated. Time complexity, as well as development complexity, also increases with the use of MARAS. Native MQTT would operate with O(1) complexity for most cases (i.e., publish/subscribe) on the client side, and O(N) on the broker side, while MARAS elevates it to O(N) on the client side and O(N^2^) on the broker side.

## 7. Conclusions

The MQTT protocol enables a fast and reliable communication scheme for IoT deployments consisting of lightweight sensors and actuator devices. But it does not natively implement any advanced security mechanism, conceivably to comply with the tight resource constraints of such devices. Yet, it is still critical to secure private data in sensitive applications. The use of SSL/TLS and HTTPS may provide significant degrees of security but at the cost of high energy consumption, low performance, exhaustive computing, and bloated bandwidth. Thus, a lightweight security protocol tailored for IoT devices with limited resources is a substantial need among the IoT ecosystems. This paper outrightly provides an extension to the MQTT protocol to further allow authorization of claimant nodes, mutual authentication of communicating parties, the privacy of topics in question, and encryption of sensitive data.

With MARAS, a Mosquitto broker can authorize the client nodes (and check their rights to publish or subscribe to specific topics) through an authentication server (WSO2IS that runs OAuth 2.0). Likewise, the nodes and the broker(s) can also authenticate each other by comparing their HMAC-based one-time passwords kept at their local storage (e.g., MongoDB). Consequently, such trusted communications can be further encrypted via AES to ensure confidentiality. This also inherently provides integrity protection. Thus, comprehensive security can be brought to the lightweight IoT devices that use MQTT. Previous works could not achieve the level of security that MARAS grants without utilizing SSL/TLS and HTTPS, which are heavy and costly.

In practice, the cost of implementing MARAS onto a sufficiently long-lasting sensor network is mainly doubling the existing data traffic in the network. This is mainly caused by the 90.74% increase in the size of PUBLISH messages, as indicated in Table 10. When the number of PUBLISH messages sent within the network exceeds 1000, they start to account for more than 99% of the cost increase caused by MARAS. Nevertheless, this extra load is still non-significant for many WSN or IoT scenarios, and the delays are very small compared to what SSL/TLS or HTTPS would cause. Additionally, the solution is not solely dependent on the technologies mentioned in this paper, yet it can also be implemented using their alternatives (e.g., RabbitMQ instead of Mosquitto). Future works will include compatibility with decentralized record-keeping systems, support for multi-broker networks, and other means of security (e.g., availability and non-repudiation).

MARAS has undergone testing on advanced devices, and our upcoming study aims to assess the feasibility of implementing our developed MARAS approach on a security-enhanced multi-protocol gateway. This gateway will incorporate BLE, Wi-Fi, and ZigBee communication protocols. To accomplish this, Mosquitto will be deployed on a Raspberry Pi and configured to support various communication technologies with ESP32 devices. This expanded testing will allow us to evaluate the effectiveness and compatibility of the MARAS approach within a diverse IoT ecosystem involving different communication protocols. In addition, the MARAS approach cannot prevent physical attacks. For this, additional security is aimed at controlling the received signal strength indicator (RSSI) information of the devices and the indoor location, and by detecting the device that does not belong to the application. Other future works may include compatibility with decentralized record-keeping systems, support for multi-broker networks, and other means of security (e.g., availability and non-repudiation).

## Figures and Tables

**Table 1:** Symbols and abbreviations.

Symbol and Abbrev.	Remarks
HOTP ( )	HMAC-based one-time password function.
*C*	The counter used in the HOTP generator.
*K*	A unique secret shared among clients and brokers.
h( )	Hashing function.
HMAC	Hashed message authentication code.
HMAC_SHA-1 ( )	HMAC function utilizing secure hash algorithm 1 (SHA-1).
JSON	Javascript object notation.
*N*	The counter setting the length of hash chains.
*p*	Hash chain string.
QoS	Quality of service in MQTT.
Truncate ( )	Truncating function.
WSO2IS	WSO2 identity server.

**Table 2:** The registered client information on WSO2IS.

Client #	Scope	client ID	Client Secret
*1*	Publish and subscribe temperature	mfsEQkwtfKINk0npCSWRb66l6jIa	1xlZwKPW6OHlGTYg1bw4lM0QfQwa
*2*	Publish temperature	i6dTy1cYXSWmEvdRGOxgaJ2f8rca	mJnxJf6bKn41Dxx6YnRoESRXwEoa
*3*	Publish and subscribe humidity	q5BMH5jPP0c3VK_SBm9fdCGuUfIa	mhMfIWt4BkUHuV0VBdhQ5ZHx_84a
*4*	Subscribe temperature	01F3krL2tXHtmt55cowbv93dlBoa	5kK7XjmQf0RDOKgxVGQScJqT7rYa
*5*	Subscribe humidity	nSAy8NhBCPpiUwfXen5Yp0dXLqMa	40GiRwSpht3JiaTd5fDF0U36oGAa
*6*	Subscribe pressure	mTm3vL0mQCqmO4GfZMchEBlt9dga	2Iq9gGc3dd8JmfBm13Nf9QjpJioa

**Table 3:** The information that a *CONNECT* message contains.

Parameter	Value
*client ID*	“mfsEQkwtfKINk0npCSWRb66l6jIa”
*Client Secret*	“1xlZwKPW6OHlGTYg1bw4lM0QfQwa”
*AccessToken*	“607c8f8b-9a16-3663-9bfe-0f05ddf34c33”
*Nth hotp*	991565
*Username*	Access Token
*Password*	AES-256 (client ID, client secret ‖ HOTP)
*Port*	1883
*Broker IP*	193.x.x.x

**Table 4:** The deciphered text.

Client #	AES-KEY (32 Bytes)	Deciphered Text	(N + 1)th HOTP (N = 6)
*1*	mfsEQkwtfKINk0npCSWRb66l6jIa	Client1secret322924	322924
*2*	01F3krL2tXHtmt55cowbv93dlBoa	Client2secret033688	033688

**Table 5:** Client’s authorization status.

Client No.	Scope/ Byte	Parsed and Decoded Scope	Topic	Permissions	Message	Authority
*1*	54	Publish temperature	temperature	Publish (w) and Subscribe (r)	36 °C	Authenticated and authorized for both receiving and sending a message about temperature.
*2*	60	Publish and subscribe temperature	temperature	Publish (w)	36 °C	Authenticated and authorized for sending a message about temperature.
*3*	54	Publish humidity	humidity	Publish (w)	*The topic does not match, ACL is denied.*	Authenticated but not authorized for transmission message about temperature.
*4*	60	Subscribe temperature	temperature	Subscribe (r)	36 °C	Authenticated and authorized for receiving a message about temperature.
*5*	58	Subscribe humidity	humidity	Subscribe (r)	*The topic does not match, ACL is denied.*	Authenticated but not authorized for transmission message about temperature.
*6*	60	Subscribe pressure	temperature	Subscribe (r)	*The topic does not match, ACL is denied.*	Authenticated but not authorized due to having pressure as the scope in the access token.
*7*	*Invalid access token or empty username*	*There is no scope information due to invalid access token*	temperature	Subscribe (r) *	*Invalid token, denied access.*	Not authenticated nor authorized.

* Set as a publisher during experiments in Section 5.

**Table 6:** Approximate time estimates in ms for various cryptographic operations on MARAS.

Operations	Time for Client Pub/Sub (ms)	Timer for Mosquitto Broker (ms)
AES (T_E_ or T_D_)	7	7
HOTP (T_HOTP_)	0.42	0.42
Hash (T_H_)	0.35	0.35
AS Introspection API (T_AS_)	N/A	10.4

**Table 7:** Computation costs comparison in ms.

Scheme	Subscriber	Broker	Publish	Total	Time
[39]	6T_H_ + 2T_E_ (144.12 ms)	13T_H_ + 6T_E_ (411.4 ms)	6T_H_ + 3T_E_ (271.53 ms)	25 T_H_ + 9T_E_ (827.05 ms)	792 ms
[40]	2T_ECM_ + 4T_H_ (35.48 ms)	5T_ECM_ + 2T_H_ + 2T_ECA_ (94.94 ms)	3Tecm + 1T_H_ + 2T_ECA_ (60.42 ms)	10T_ECM_ + 7T_H_ + 4T_ECA_	190 ms
MARAS	T_E_ + T_HOTP_ + T_H_ (7.77 ms)	2T_D_ + 2T_HOTP_ + 202T_H_ + 2T_AS_ (106.34 ms)	T_E_ + T_HOTP_ + T_H_ (7.77 ms)	2T_E_ + 2T_D_ + 4T_HOTP_ + 202T_H_ + 2T_AS_	121.8 ms

**Table 8:** Communication costs comparison in bits.

Scheme	Number of Messages	Total Cost (bits)
[39]	7	2560
[40]	5	2560
MARAS	3	2432 over public channel 2672 over the TLS

**Table 9:** Comparative analysis of existing works and MARAS.

Literature and MARAS	Authorization	Role-Based Access Control	Client Authentication	HOTP	Mutual Authentication	Confidentiality (Payload Encryption)
Azzedin et al. [17]	✓	✓	✕	✕	✕	✕
Fremantle et al. [21]	✓	✓	✓	✕	✕	✕
Singh et al. [28]	✓	✕	✓	✕	✕	✓
Upadhyay et al. [29]	✓	✕	✓	✕	✕	✕
Niruntasukrat et al. [30]	✓	✕	✓	✕	✕	✕
Zamfir et al. [31]	✕	✕	✓	✕	✓	✓
Rajan et al. [32]	✓	✕	✕	✕	✕	✓
Nagarajan et al. [33]	✕	✕	✓	✕	✓	✓
Alshammari [34]	✓	✕	✓	✕	✓	✓
Fathy et al. [35]	✕	✕	✕	✕	✕	✓
Shilpa et al. [36]	✓	✕	✓	✕	✓	✓
Winarno et al. [37]	✕	✕	✕	✕	✕	✓
Ramyasri et al. [38]	✕	✕	✓	✕	✓	✓
Patel et al. [39]	✕	✕	✓	✕	✓	✓
Saqib et al. [40]	✕	✕	✓	✕	✓	✕
Katsikeas et al. [41]	✕	✕	✕	✕	✕	✓
Bhawiyuga et al. [42]	✕	✕	✓	✕	✕	✕
Bashir et al. [43]	✕	✕	✕	✕	✕	✓
Wardana et al. [44]	✓	✕	✓	✕	✕	✓
Calabretta et al. [45]	✓	✕	✓	✕	✕	✕
Bali et al. [47]	✕	✕	✓	✕	✓	✓
Sundarrajan et al. [48]	✕	✕	✓	✕	✕	✓
Aknin et al. [49]	✕	✕	✓	✓	✓	✕
Sanjuan et al. [50]	✕	✕	✓	✕	✓	✓
Amoretti et al. [51]	✓	✓	✓	✕	✕	✕
Blazy et al. [52]	✓	✓	✓	✕	✕	✓
MARAS	✓	✓	✓	✓	✓	✓

**Table 10:** Extra network traffic caused by MARAS (in terms of message size).

Message Type and N *		Native MQTT	MARAS	% Cost Increase	% Share in Total Cost Increase
*CONNECT*	N = 1	110 B	237 B	115.46%	70.95%
	N = 1000	110 B	237 B	115.46%	0.26%
*PUBLISH*	N = 1	54 B	103 B	90.74%	27.37%
	N = 1000	54 kB	103 kB	90.74%	99.74%
*SUBSCRIBE*	N = 1	17 B	20 B	17.65%	1.68%
	N = 1000	17 B	20 B	17.65%	0.01%

* N solely indicates nr. of data packages to be published in entire network lifetime.

## Data Availability

No new data were created or analyzed in this study. Data sharing is not applicable to this article.

## Supplementary Materials

The following supporting information can be downloaded at: srg.cs.deu.edu.tr/publications/2023/maras/ (accessed on 2 May 2023).
